# Closing the Integration Gap: A Pilot for Incorporating Foundational Sciences, DEI-Decision Making, Empathy, and Communication for Congestive Heart Failure and Arrhythmia Management by Pharmacy Students

**DOI:** 10.3390/pharmacy10040077

**Published:** 2022-07-01

**Authors:** Ashim Malhotra, Song Oh, Zhuqiu Jin, Xiaodong Feng

**Affiliations:** 1 Department of Pharmaceutical and Biomedical Sciences, California Northstate University College of Pharmacy, Elk Grove, CA 95757, USA; zhuqiu.jin@cnsu.edu; 2 Department of Clinical and Administrative Sciences, California Northstate University College of Pharmacy, Elk Grove, CA 95757, USA; soh@sju.edu (S.O.); xfeng@cnsu.edu (X.F.)

**Keywords:** cardiovascular diseases, arrhythmia, congestive heart failure, pharmacy education, diversity, equity, inclusion, teaching empathy and communication

## Abstract

Pharmacists must integrate foundational sciences with pharmacy practice for providing optimal patient care. Pharmacy students need to be trained to provide culturally competent, linguistically accessible, and empathetic care while integrating foundational science principles. However, such holistic integration is challenging to achieve and assess. To bridge this gap, we implemented and assessed an “integrated cardiovascular simulation” (ICS) module for P2 students, employing case-based and team-based learning. ICS focused on congestive heart failure with preexisting arrhythmia and incorporated patient counseling relating to diversity factors such as cultural competency, linguistic challenges, and the impact of population diversity on cardiac diseases. Students learned the SBAR communication technique (situation, background, assessment, and recommendation) and recommended therapy while elaborating on drug MOA and adverse effects. ICS was assessed through pre-and post-session quizzes and perception data immediately after the activity, and after two years, when students progressed to the cardiovascular APPE block. Student performance improved on a post-test (80.2%) compared to the pre-test (66.9%), *p* < 0.01 paired student *t*-test, with an increase in symptom and arrhythmia pattern recognition (41.2% and 36.7%, respectively). ICS was effective for teaching (1) arrhythmia pathophysiology (85%), (2) EKG interpretation (89%), (3) drug adverse effects (93%), (4) DEI-clinical decision making (92%), and (5) communication skills (85%).

## 1. Introduction

Foundational sciences are an integral part of health professionals’ education. Ever since the publication of the seminal 1910 Flexner Report [1], foundational sciences are placed early in the medical education curriculum to maximize learners’ ability to apply these principles in the evaluation and management of diseases. This “traditional” approach contextualizes clinical decision-making in a foundational sciences reference frame and has been extended to other health professions education programs such as pharmacy education [2]. Indeed, studies have shown that integrating foundational and clinical curricula achieves the aim of integrating foundational sciences with their clinical application by motivating adult learners [3,4,5]. Nevertheless, effective integration strategies and approaches that will help create meaningful, higher-cognition, and collaborative learning experiences to engage learners in enjoyable, real-world problem solving remain an urgent need [6].

To bridge this integration gap and design a “hands-on” collaborative team learning experience we designed, implemented, and assessed our “Integrated Cardiovascular Simulation” (ICS) program to encourage second-year students (P2) in our four-year Doctor of Pharmacy (PharmD) program to synthesize and apply cardiovascular pathophysiology and pharmacology to the clinical decision-making process.

Although integration strategies are documented in the pharmacy education literature, albeit sparingly, integration remains difficult to achieve. Many factors contribute to this, though none as remarkable as a lack of concrete “how-to” examples of effective integration activities [7,8]. Hopkins et al. reported that despite “apparent will”, lack of integration ailed pharmacy education, citing resistance to change by faculty as a paradox causing “change without effective changes” [9]. Overall, integration remains difficult to achieve and measure, creating an urgent need for engaging, learner-centered integration programs.

Furthermore, pharmacy students experience a challenge transitioning their knowledge from the foundational sciences to pharmacy practice and clinical experience [10]. Anecdotally, this manifests as a “single correct answer problem”. Our colleagues will recognize that students in the P2 year are often confused when faced with the idea that multiple therapeutic options may be available for a patient and there is not a “single” correct answer. Rather, students need to recall and synthesize foundational sciences information and apply it with critical thinking to that specific patient case. It is our opinion that at its fundamental basis, this is a problem originating from a lack of students’ ability to integrate foundational science information with its practice application in the clinical decision-making process.

Additionally, delivering patient care in the US requires health care providers to develop several “soft skills”. For example, the 2013 CAPE Outcomes outline aspirational goals that pharmacy students should be able to demonstrate, such as achievement of cultural competency, the ability to educate patients and their families while providing empathetic care, and overcoming linguistic challenges [11]. However, seldom do didactic activities afford opportunities for students to learn these difficult skills that require practice in a safe and positive environment, such as pharmacy school. An advanced high-fidelity simulation offers many opportunities to embed cultural competency, diversity, and empathy training while teaching strategies to overcome linguistic barriers when working with patients and their families.

Racial, ethnic, and gender diversity are known risk factors for cardiovascular diseases, such as heart failure [12]. The prevalence of heart failure is higher among African Americans and Hispanics when compared to Whites, and highest among African American women compared to any other group in the US [13]. Health care providers need to not only be aware of these risk factors when providing care, but they also need to be trained to pro-actively provide culturally sensitive, linguistically accessible care with adequate opportunities for communication through the utilization of effective and proven communication techniques.

However, students may find it a challenge to simultaneously deal with providing culturally competent and empathetic care and practicing communication techniques, while learning to integrate the cardiovascular pathophysiology and pharmacological basis of clinical decision making, especially for the first few times. To address this challenge, ICS engaged P2 learners to begin to connect the pharmacotherapeutic management of congestive heart failure (CHF) and arrhythmia, emphasizing the pharmacological basis of clinical decision making while incorporating select elements related to empathy, cultural competencies, such as a linguistic barrier, patient history taking, and communication strategies such as the “SBAR” technique.

## 2. Materials and Methods

### 2.1. Need for ICS

**Figure 1:**
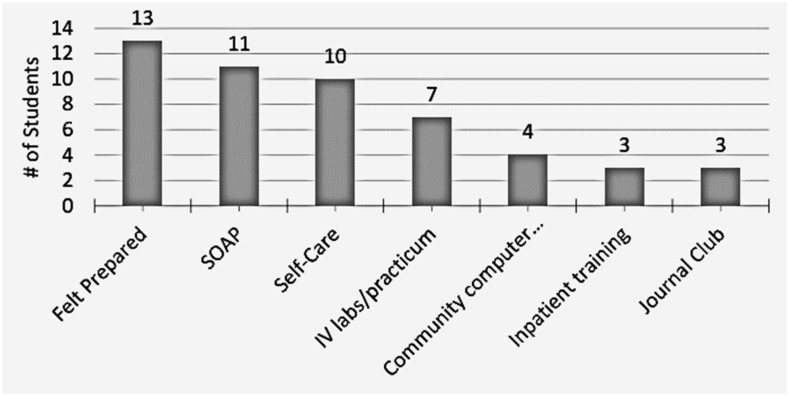
Survey results from a quality control student perception survey conducted by the Department of Experiential Education for students enrolled in the cardiovascular APPE regarding the perception of their confidence and level of preparation for different aspects of the APPE rotation. The Y-axis lists the number of students who expressed confidence in their ability to perform specific tasks during the ambulatory care APPE with a cardiovascular focus.

The California Northstate University College of Pharmacy (CNUCOP) regularly conducts institutional research in the form of student surveys to gauge the implementation, impact, and success of various initiatives and programs. In the academic year 2018–2019, while conducting such a survey, the Experiential Education Department asked students enrolled in the final year of our Doctor of Pharmacy (PharmD) program undertaking their first Advanced Pharmacy Practice Experience (APPE) about their perceived degree of preparation for hospital APPE rotations, and in particular, for providing care for hospitalized patients with cardiovascular diseases. As shown in Figure 1, of the 14 student respondents enrolled in the ambulatory care APPE with a cardiovascular focus, only 3 students felt confident about providing in-patient care. Similar results were obtained (not shown) in surveys of P2-year students regarding self-perceptions of preparedness for their in-hospital Introductory Pharmacy Practice Experiences (IPPE). The current ICS feasibility pilot program was birthed directly out of a need to close the student-perceived gap in their ability to integrate the foundational science context of cardiovascular disease states at the introductory level in a hospital setting.

### 2.2. Curricular Design

The ICS study reported here was designed to enhance student confidence in clinical decision-making and to improve their comfort level with the workflow in a hospital setting, including handling the pressures of working as a pharmacist in a US-based hospital, while integrating their foundational sciences knowledge with clinical decision making and the pharmacist patient care process (PPCP).

ICS was designed as a cross-sectional, single-blinded feasibility pilot, the impact of which was evaluated using a mixed-methods approach. The corresponding author, who served as the PI, was blinded to the scores received by the students in all assignments. ICS was approved by our University’s IRB (IRB# 2001-01-64) and was conducted in person in the fall of 2019, immediately before the onset of the global COVID-19 pandemic.

**Figure 2:**
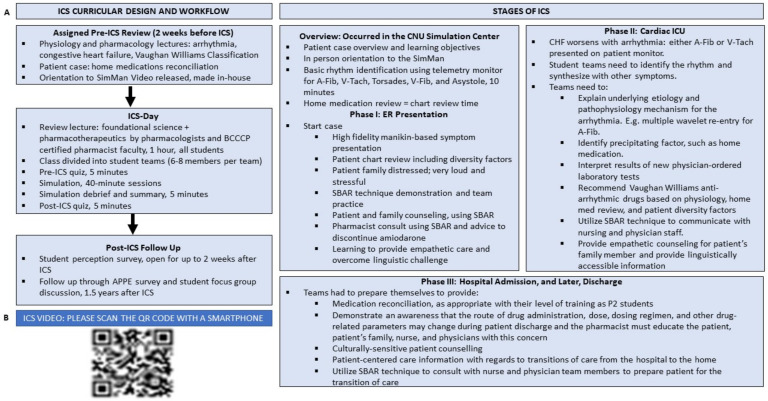
Over of ICS and Video Showcasing the Activity. (**A**) The schema depicts the overall curricular design for ICS. ICS was placed in the second professional year of our Doctor of Pharmacy program to help students connect the dots between the pathophysiology and pharmacology of congestive heart failure (CHF) and various arrhythmias with the clinical decision-making process. Students were oriented to ICS and its goals and objectives ahead of time, and lectures in the regular and required cardiovascular pathophysiology and pharmacology course were assigned as pre-reading. ICS employed an amalgamation of Case Based Learning, Team Based Learning, and high-fidelity simulation. Advanced Cardiac Life Support (ACLS)-type emergency cardiac patient cases were developed and student teams of 8–10 members worked together in 45-min-long sessions to identify the cause of the arrhythmia and CHF, recognize the arrhythmia, provide patient counseling, communicate with other healthcare providers using SBAR, and eventually practice medication reconciliation. ICS was assessed using pre and post-tests and a learner perception survey. (**B**) A QR code is provided that links the reader to a video depicting a part of the ICS. Please point a smartphone camera at the QR code and follow the link.

ICS included 86 students from the graduating Class of 2022 who were at that time enrolled in the second professional year (P2) of our accredited four-year PharmD degree program. Our class size is approximately 100 +/− 20 students. We carefully placed ICS before the pharmacotherapeutics course in the spring of the P2 year to enable students to practice how the concepts of the foundational sciences connected with the clinical decision-making process so that they were more engaged when they encountered this content in that course. Figure 2 depicts the overall scheme and important points in ICS design. ICS was placed in our skills-based “practicum” laboratory course called “PRC709”. The primary goal of ICS was to help P2 pharmacy students connect the dots between the didactic cardiovascular pathophysiology and pharmacology P2 course called “PBS701: Pathophysiology and Pharmacology II-Cardiovascular” and its clinical application through patient-centered problem solving and critical thinking. The PRC709 and PBS701 courses run concurrently in the fall semester of the P2 year, thus allowing horizontal integration for the cardiovascular content.

To achieve this goal, we developed two realistic Advanced Cardiovascular Life Support (ACLS) patient cases. The patient cases were operationalized using high-fidelity manikin-based simulations and trained “empathy” actors at our University Simulation Center. ICS employed Case-Based Learning principles and focused on CHF with preexisting arrhythmias as comorbidity.

### 2.3. Grounding ICS in Educational Theory

Our active learning strategy was grounded in the adult theory of education, which posits that adults learn better when they perceive that their educational plan is meaningful and useful [4,5]. Our pedagogy approach is grounded in the educational theory called “constructivism”, which states that learners learn better through active engagement and participation. Learners progressed through the stages described in the educational theory/model known as Miller’s Pyramid of Clinical Competency: from “knowing” to “knowing how” to “showing” to “doing” (based on constructivism).

Although simulation has been employed in medical education [14], pharmacy programs have only recently begun to incorporate simulation in the PharmD curriculum. Even so, simulation in pharmacy education remains restricted to skills training programs ranging from introductory to Basic Life Support (BLS) training [15]. However, the use of mid-and high-fidelity manikins remains more common in medical education. The significance of our approach for integrating foundational sciences with clinical decision-making was captured in the (1) curricular design, (2) curricular placement, (3) patient case development, (4) debriefing sessions, (5) flexibility, and (6) the amalgamation of Case Based Learning (CBL), Team-Based Learning (TBL), and high-fidelity simulation for integration.

### 2.4. Case Based Learning: ACLS Patient Cases Play-Acted through Simulated Hospital Stages

ICS exemplifies collaboration between pharmacologists and clinical pharmacy faculty specializing in cardiovascular diseases. A board-certified critical care pharmacist (BCCCP) with postdoctoral training in acute critical care spearheaded the development of two “real-life” patient cases based on published case reports. Another pharmacy practice faculty member with expertise in cardiovascular care reviewed the cases. Patient cases were discussed with pharmacology faculty to incorporate the “learning objectives” presented in the cardiovascular foundational science course taught in the fall of the P2 year, PBS 701.

In our teaching, we have experienced that learners need to practice linking foundational science information to real-life disease state management, marking the true beginning of integration. To achieve this goal, we carefully linked the learning objectives of lectures in PBS 701 with learning outcomes for ICS. Lectures selected included (1) electrophysiology of the heart, (2) cardiac rhythm, (3) arrhythmias, (4) antiarrhythmic drugs, (5) CHF, and (6) select CHF medications.

### 2.5. Workflow and Activity Implementation

Prior to ICS, all P2 students attended the foundational science lectures listed above and took quizzes in the didactic course. In preparation for ICS, students were reassigned these lectures as pre-reading in our TBL pedagogy. Patient cases were released two weeks ahead of ICS using the Learning Management System Canvas. Patient cases included information using standard terminology found on real-life hospital forms, such as chief complaint, history of present illness, past medical history, allergies, family history, social history, and a “home medication” list. Please see Figure 2 and Table 1.

### 2.6. Designing the Two Patient Cases

#### 2.6.1. Patient Cases’ Differences and Similarities and Study Plan

We developed two different clinical scenarios as two separate patient cases. Patient case-1 focused on the presentation of CHF with drug-induced, hyperthyroidism-based atrial fibrillation. As the case progressed from emergency room (ER) presentation (phase I) to hospital admission (phase II) to discharge (phase III), the case became increasingly complex. For example, a few hours into Phase I, we simulated physician-ordered laboratory tests. Student teams had to factor in the laboratory values in their analysis to determine that the cause of the atrial fibrillation being projected on the telemonitor was drug-induced, for which they needed to recommend discontinuation of amiodarone, which contains Iodine. Each of the phrases in the previous sentence had to be verbalized by the student team to demonstrate their thought process. Please refer to the detailed Learning Objectives outlined in Table 2.

The faculty facilitated and helped each of the student teams proceed in a stepwise manner. For example, each of the student teams was prompted to state (1) the medicinal chemistry information about amiodarone (that it contains Iodine), (2) amiodarone’s Vaughan Williams classification, (3) the interpretation of laboratory values of blood hormone levels, and (4) that the cardiac rhythm being shown represented atrial fibrillation (in this case). Furthermore, teams were also taught to use the SBAR communication technique, as detailed below. Teams needed to interact with an actor who role-played a very emotional family member who presented great stress.

In the second clinical scenario (patient case-2), although the demographic, family history, and the rest of the patient profile remained the same, the simulation included ventricular tachycardia, ventricular fibrillation, and Torsades de pointes due to a change in potassium level, which was reflected in the laboratory values. The two clinical scenarios were played out one after the other. The faculty announced that the two cases were different and drew students’ attention to the absence of the T_3_/T_4_ hormone values for patient case-2.

Both patient cases were released two weeks before the commencement of ICS. Both patient cases were role played within the total allotted 45 min for each simulation session for one batch of student teams. The CNUCOP Academic Affairs Office assigns students to teams at the beginning of each semester. Each student team is composed of up to six students. The posted patient cases, along with the lecture material of the concurrent PBS701 foundational sciences course in cardiovascular diseases, pathophysiology, and pharmacology contain pre-reading assignments. Following the TBL method, students answered an initial quiz called an individual readiness assurance test (iRAT) based on the pre-reading. Students took this quiz again at the end of the simulation. Due to time limitations, these pre-and-posttests constituted the primary content retention measure.

Patient cases were operationalized using the Laerdal SimMan 3G high-fidelity manikin, an actor role-playing the part of the family member and a faculty member role-playing a physician. All faculty were trained and participated in at least 3 h of discussion of the case and workflow. The study PI (pharmacologist) and the BCCCP pharmacist faculty members facilitated the case. They also asked verbal questions that were pre-written as they presented each clinical scenario. The questions prompted student teams to continually embrace the various aspects of the unfolding case. Student responses to these questions were corrected and facilitated by all faculty. However, these questions were not graded.

Phase I—Emergency Room (ER) Presentation. ICS commenced with a patient presenting to the simulated ER—the SimMan was programmed to cough, wheeze, and have difficulty breathing, a bilateral leg and ankle edema, carotid bruits, a history of recent syncope, palpitations, and hyperthyroidism, and an S3 ventricular gallop. Eighty-six P2 students divided into 8–10 membered teams reviewed home medications to identify potential drug causes, after correctly identifying each symptom and diagnosing CHF. Subsequently, the faculty reviewed the SBAR communication technique: “Situation, Background, Assessment, and Recommendations”. Student teams were required to formulate a recommendation including stopping or starting medication, drug mechanism of action (MOA), and adverse effects learned in PBS701, and to communicate their intervention to the “medical team” using SBAR. Phase I also included a metabolic laboratory panel with calcium, magnesium, complete blood count, thyroid function test (TFT), and INR to determine the cause of the arrhythmia. Specifically, we provided an abnormal TFT indicating hyperthyroidism. Faculty instructors facilitated discussions on home medication-induced (amiodarone-induced) hyperthyroidism and atrial fibrillation.

Phase II—Cardiac Intensive Care Unit (ICU) Presentation.

This phase focused on the pathophysiology of arrhythmias and the pharmacology of antiarrhythmic drugs. SimMan was programmed and telemetry was used to present atrial fibrillation, ventricular tachycardia, Torsades de Pointes, ventricular fibrillation, and asystole. Student teams learned to identify each arrhythmia, with faculty facilitation, and explain the altered EKG. Upon arrhythmia identification, faculty instructors facilitated discussions on potential treatment based on Vaughan Williams’ classification, reinforcing foundational sciences learning and application. Additional laboratory results depicting new electrolyte disturbances were provided. Specifically, for ICS, furosemide-induced hypokalemia and hypomagnesemia eventually led to QTc prolongation and Torsades de Pointes.

A novel aspect incorporated in phase II was significant emotional distress exhibited by a family member (staff actor). Although some students prioritized patient and family member interactions, many were not quite sure how to react to the difficult encounters. Faculty instructors reminded students to ensure communication to provide patient-centered care.

Phase III—Hospital Discharge

After several days of “hospital stay”, the patient was ready for discharge. Phase III focused on medication reconciliation and patient counseling. Although P2 students had beginner’s knowledge of pharmacotherapeutics, we emphasized considering the overall clinical course and identifying appropriate changes that should be made by comparing the home medications and the current medications to bridge extant experience with clinical decision-making skills.

A second patient case was also presented. For the second case, the patient profile including the Chief Complaint, History of Present Illness, Past Medical History, Family History, Medications, and Allergies remained the same. The following laboratory values after the presentation to the ER, specifically, K 3.1 mEq/L were changed, however.

#### 2.6.2. Session Planning

It took six three-hour-long meetings (18 contact hours) between the two principal faculty (pharmacologist and critical care pharmacist) to plan ICS in the summer of 2019, including linking PBS701 learning objectives. Additional patient case reviews took one contact hour. ICS was incorporated into our P2 practicum course (PRC709) and took eight hours of class time, commencing with an in-class lecture by the critical care pharmacist, a pre-test on Canvas, followed by 45-min-long simulation sessions repeated throughout the day, with each session concluding with a debriefing and a post-test. A staff member was trained as a family member actor, whereas another staff member filmed ICS. Faculty received extensive training (3 months) in programming simulations using the high-fidelity SimMan.

#### 2.6.3. Student-to-Faculty Ratio

ICS can be operationalized with two faculty for about 90 students divided into 8–9 teams on an 8 am–5 pm schedule. Small teams are critical for effective debriefing. A pharmacy resident or staff member can help with coordination, logistics, attendance, and emergent issues.

### 2.7. Quizzes, Survey Administration, Data Collection, and Statistical Analysis

We designed a multiple-choice question-based pre-and-posttest that included elements of the foundational sciences content of ICS. We based the design of this test loosely on the 2012 work of Harris et al. who employed a high-fidelity simulator to teach medical students cardiac function curves [16]. For example, similar to their approach we included six questions in the pre-and-posttest. Each student individually took a pre-test and post-test immediately before and after the ICS, respectively. Due to the time limitations of conducting ICS for multiple student teams within the total allotted 8-h-long timeframe in this practicum course, the number of questions that could be included in this pre-and-posttest was limited (please see the limitations section below). Questions are listed in Table 3. A total of five minutes out of the total 45 min for each session were allocated for each test. The tests were paper-based, and a faculty member other than the PI collected, graded, and entered the test scores in our learning management system, Canvas. Prior to the release of the scores on Canvas, statistical analysis was conducted using a single-blind approach.

In addition to the in-session pre-and-post content quizzes, we also developed and administered a student perception survey based on Harris et al. [16]. However, we expanded the survey, and our instrument comprised of 15-items on a 5-point Likert scale and was administered through SurveyMonkey. The responses were completely anonymous. The survey items along with an analysis of student responses are presented in Table 4.

For statistical analysis, we used a paired, two-tailed Student *t*-test for the pre-and-post quizzes, with an alpha value of *p* < 0.05. The PI was blinded to student identities throughout the study.

## 3. Results

### 3.1. Content-Based Pre-and-Post Tests

The mean percentage of correct assessment responses increased from 66.9% in the pre-test to 80.2% on the post-test with a change in score of 13.3% (*p* = 0.01; two-tailed paired student *t*-test); see Table 3 for a detailed analysis.

### 3.2. Student Perception

As shown in Table 3, we also analyzed student performance by categorizing the quiz questions per the content area. We found that performance improved on questions regarding recognition of heart failure symptoms (41.2%) and arrhythmia patterns (36.7%). Furthermore, students felt that ICS helped them learn: (1) arrhythmia pathophysiology (85%), (2) EKG interpretation of arrhythmias (89%), (3) adverse effects of antiarrhythmic medications (93%), (4) clinical decision making (92%), and (5) communication skills between team members (85%).

Table 4 presents the results of the student perception survey, categorized into the three broad areas (1) impact of ICS on learner’s ability to integrate foundational sciences with clinical decision making, (2) student perception of the learning experience, and (3) the overall impact of ICS on learning. We received responses from all participating students. For example, 91% percent of the respondents felt that ICS made the content more clinically relevant than lecture, whereas student perception of their interaction with the simulated patient was rated at 74%. Although most of the questions performed well, the following three areas for improvement were identified based on the survey responses: a greater need to (1) integrate the underlying pathophysiology of arrhythmias (area 1), (2) increase the in-session training time for rhythm identification and SBAR practice (area 2), and (3) slow the pace of ICS (area 3).

### 3.3. Evaluating the Longer-Term Impact of ICS

Finally, to further evaluate learner perception regarding overall confidence in the application of concepts acquired during the simulation to real-life scenarios, we surveyed student perceptions of their preparation for providing patient-centered care during relevant APPE rotations. This APPE survey was conducted as a regular part of our curricular quality control and students were asked to rate their confidence and preparedness for APPE rotations, SOAP noting, and the provision of essential pharmacist services for patients suffering from cardiovascular diseases when compared with their abilities for the same for inpatient pharmacy or other services. Eighty-one percent of students indicated that they felt more prepared for cardiovascular block APPE rotations, with qualitative responses suggesting a significant role for simulation (data not shown). In 2022, the Curriculum Committee called a student focus group discussion for P4-year students to identify which curricular improvements resulted in the enhanced learning experiences and students identified ICS as an effective technique that made them learn about providing care for a cardiovascular patient in the hospital setting (verbal communication to the PI).

## 4. Discussion

### 4.1. Framing and Contextualizing ICS

High fidelity simulation has been effectively employed in medical education, though it is infrequently used in pharmacy education. The central goal of ICS was to increase students’ familiarity and knowledge of cardiovascular patient case management in the hospital setting. Although at some hospitals, pharmacists may not be by the bedside in cardiac emergencies, the roles of pharmacists in ACLS care, especially for dosimetry calculations, is an important opportunity. Typically, in small hospitals, nurse providers are relied upon to conduct complex dose conversion calculations, and literature has documented a high rate of medication errors [17,18,19], which may be prevented by having pharmacists perform these functions.

Based on the student perception data, overall ICS was successful in all three areas of enhancing students’ ability to integrate foundational science knowledge with clinical decision making, augmenting the pre-IPPE learning experience, and impacting students’ learning. Respondents indicated that ICS helped them learn: (1) arrhythmia pathophysiology (85%), (2) EKG interpretation of arrhythmias (89%), (3) adverse effects of antiarrhythmic medications (93%), (4) clinical decision making (92%), and (5) communication skills between team members (85%). Ninety-one percent felt that ICS made the content more clinically relevant than lectures, whereas student perception of their interaction with the simulated patient was rated at 74%.

Because we based our student perception survey on previously published work by Harris et al., we did not independently validate the survey. However, because we expanded the survey, in the future as we plan to expand ICS, we will also validate the survey.

### 4.2. Closing the Literature Gap: Comparison of ICS with Cardiovascular Teaching Simulations

Although high fidelity simulation and other in-person and virtual simulations have been reported in the pharmacy, medical, and nursing education literature, ICS significantly differed from this body of work in curricular design, intentionality, implementation, and assessment.

For example, Bose created an arrhythmia-focused teaching activity for P2 students at the Western New England University School of Pharmacy that included EKG rhythm identification [20]. He later expanded this into a P3 elective where students were allowed to practice rhythm identification using a high-fidelity manikin. However, ICS differed from this curricular design significantly because we integrated foundational science background and clinical decision-making simultaneously and used the manikin and the patient family actor for empathy and communication training.

Moreover, some studies have reported the use of virtual computerized patients to simulate cardiovascular disease state presentation instead of the comprehensive high-fidelity manikin. For instance, at the University at Buffalo School of Pharmacy and Pharmaceutical Sciences, Woodruff et al. [21] created a heart failure virtual patient simulation using Adobe Captivate 2019 Release (Adobe System Incorporated, San Jose, CA, USA) in a required pharmacotherapy course to enhance heart failure-related pharmacotherapy understanding for P2 students. Although this was not a high-fidelity simulation, students were presented with heart failure-related aspects by a virtually simulated patient and an automated algorithm that redirected students to specific parts of the simulation if related questions were incorrectly answered.

Similarly, Douglass et al. [22] argued that high fidelity manikins add a prominent cost factor in designing active learning modules for training pharmacy students to provide cardiovascular patient care. Therefore, they demonstrated the use of a virtual simulated patient platform in improving P3 students’ post-test scores in the advancement of drug-therapy-based clinical skills and management of heart failure.

Interestingly, although over the past decade, pharmacy programs across the United States have used high fidelity simulation in teaching cardiovascular diseases, these teaching programs mainly focused on improving knowledge of advanced cardiovascular life support (ACLS) and student confidence and satisfaction [23,24,25,26,27]. Additionally, assessment of communication with other healthcare providers using high fidelity simulation is still scarcely reported in pharmacy education, with inconsistent findings [27,28].

Importantly, however, despite the consistent reporting of enhanced student reception of simulation, knowledge improvement as measured by post-assessment scores is challenging [15,23]. We found a similar challenge in adequately and quantitatively capturing students’ knowledge gains in specific content areas based on ICS. For example, only two of the questions on our pre-and-post test showed improvement. Following a detailed discussion and analysis of the available literature, we have learned that augmenting our assessment strategy may be a useful approach. We are currently working on video capturing the verbal question-and-answer section, the viva portion of ICS that occurred during the simulation. In future iterations of ICS, we hope to provide this recorded video interaction to student teams for further review, self-assessment, and self-directed learning, while in addition using this to score team performance. We are developing a rubric that will be used to guide faculty scoring of team performance.

In our simulation, students were encouraged to use the SBAR technique to facilitate communications with the patient’s physician and nurse. To our knowledge, this is a current gap in the pharmacy high fidelity simulation and integration literature which ICS addresses for the first time. Approaches similar to ICS may be gainfully employed to teach and assess pharmacy students’ development of skills to provide empathetic, linguistically accessible, and culturally sensitive patient care while focusing on diversity-related patient demographic and risk factors for cardiovascular cases.

Although Marken et al. [29] reported the use of human simulators and standardized patients to teach students to navigate difficult conversations, their simulation focused on interprofessional education with interdisciplinary teams. Lastly, the placement of high-fidelity simulation within the curriculum is a notable difference between the previously published studies and our study. Most, if not all, previously implemented simulations are placed during or after the pharmacotherapy course. ICS was unique in that it was placed before students learned pharmacotherapeutic management of cardiovascular diseases. Despite having only the foundational science course, students were challenged to develop therapy plans that are specific to the patient’s case.

### 4.3. Resources for Implementation

ICS required (1) faculty time from a cardiovascular pharmacologist and ACLS-trained critical care pharmacist, (2) costs of a Laerdal SimMan 3G manikin ($200,000), an operational simulation center ($685,000), and its maintenance ($58,000 per annum), (3) SimMan training, (4) curricular flexibility, (5) access to a learning management system, and (6) administrative support. Costs for high fidelity manikins may be partially offset by collaborating with medicine, nursing, or physician assistant programs. For our ICS, faculty and staff time were in-kind contributions ($8100 at $100 per hour for 27 total hours). Although the costs may seem daunting, many universities in the US, especially those with nursing programs, house full-scale simulation centers, and collaboration may be a way forward.

### 4.4. Transferability to Other Programs

When incorporating ICS, the sequence of the curriculum must be examined to determine appropriate placement. To achieve its main goal of bridging the integration gap between foundational and clinical sciences, ICS should be placed after the cardiovascular pathophysiology and pharmacology courses and before the pharmacotherapeutics course. If available, a pharmacy skills laboratory course would be an ideal place to incorporate ICS. Collaboration between foundational science and clinical faculty is critical for developing a meaningful ICS patient case. Identifying dedicated faculty to consistently lead the simulations between each student group is important to enhance the consistency of in-simulation and the debriefing discussions.

### 4.5. Limitations

Our study was limited by the small student sample size and a single student cohort. The pre-and post-assessments were completed on the day of the simulation and there was no follow-up to assess long-term retention. Additionally, we learned from this pilot study that the number of questions to assess student performance needs to be higher. However, at our college, this would need a substantial time commitment to increase the curricular time afforded to ICS. Already, one entire day had to be set aside for ICS during PRC709 and a special dispensation was needed from the Dean’s Office to create this curricular mechanism. For the future, we have currently initiated a conversation about considering mechanisms to increase the time dedicated to ICS in the Curriculum Committee. We are considering splitting the student teams and conducting the activity thrice over three afternoons. Furthermore, although we measure horizontal integration directly through the pre-and-post quizzes and students’ perceptions, and indirectly through the in-session viva voce verbal session if more time is dedicated to ICS, we acknowledge that this assessment would benefit from it.

In addition, the enhancement of communication skills may be achieved by incorporating diverse patient and family interactions. Although we relied on the trained actor to provide cues for emotional feedback and diversity and inclusion-related aspects, expanding this section will enhance ICS. Faced with difficult encounters, students will develop effective communication skills including empathy while providing patient-centered care. Based on the findings from this study, the assessment questions can be further evaluated for validation. Commonly missed points should be evaluated for the potential gap in student understanding and used to improve teaching points during the simulation.

## 5. Conclusions

ICS demonstrated that high-fidelity, pharmacology-based simulations which are horizontally aligned and integrated with concurrent curricula may be effectively applied to teach foundational science-premised clinical decision-making in complex cardiovascular care. Incorporating linguistic and cultural competency through case-specific diversity, equity, and inclusion factors augmented the learning experience by engaging our adult pharmacy learners. Our data indicate that constructivist learning practices such as the ICS module enabled pharmacy students to practice communication strategies for complex social interactions. There was a measurable enhancement of the internalization and retention of the ability to integrate the many aspects of the pharmacist patient care process. Although the rationale for the current cardiovascular cases was to help students synthesize the knowledge they had thus far acquired regarding the etiology, pathophysiology, and foundational pharmacology for treating congestive heart failure and arrhythmia, an ICS-like approach may be adopted for teaching other disease states. We are currently planning on designing a similar high-fidelity manikin-based simulation for teaching the clinical management of chronic kidney disease in the context of linguistic challenges and a need for the provision of culturally sensitive care. Future studies in the field focused on developing and standardizing rubrics for teaching and assessing empathy and communication strategies, and students’ incorporation of patients’ diversity-related factors will help expand this important educational area. In conclusion, ICS is an effective, engaging, and “hands-on” learning strategy for incorporating age, gender, and racial and ethnic diversity-based clinical decision-making.

## Figures and Tables

**Table 1:** Patient Case 1. CHF, Drug-Induced Hyperthyroidism, and Related Arrhythmia.

Phase-I		
Chief Complaint: “Lately, I feel like my heart has been racing a bit. It really doesn’t bother me that much, but I wanted to have it checked out to be sure.”		
History of Present Illness: Cooper Riley is a 64-year-old Black male with heart failure and a history of persistent AFib who presents to his primary care physician complaining of palpitations that he first noticed 7 days ago. He reports that he is aware of the palpitations but that he has remained relatively asymptomatic. There has not been a noticeable change in his level of fatigue or exercise capacity during his normal daily activities. Mr. Riley has had congestive heart failure for 6 years. For the past few years, his baseline exercise capacity would be described as a slight limitation of physical activity with some symptoms during normal daily activities but asymptomatic at rest. He has a history of AFib that was cardioverted to NSR and he has been on amiodarone to maintain NSR for the past 8 months.		
Past Medical History		
Hypertension Persistent AFib (previously in NSR with amiodarone therapy) Heart failure with reduced ejection fraction (LVEF 35%) Obstructive sleep apnea (AHI 28 events/hour), alleviated with CPAP therapy		
Family History: Both parents are deceased. His father died from AMI at age 64. His mother died of breast cancer at the age of 70 years.		
SH: Mr. Riley works as an accountant. He is married with two healthy children. He does not smoke but occasionally “drinks a few beers on the weekend.” His wife, who is accompanying him, only speaks Spanish.		
Medications		
Carvedilol 6.25 mg PO BID Digoxin 0.0625 mg PO once daily Amiodarone 400 mg PO once daily Furosemide 40 mg PO once daily KCl 20 mEq PO once daily Lisinopril 10 mg PO once daily Warfarin 5 mg PO once daily		
Allergies: No known drug allergies		
Phase-II-Laboratory Values		
Na 140 mEq/L	Hgb 12.0 g/dL	Ca 8.5 mg/dL
K 4.0 mEq/L	Hct 35.8%	Mg 2.1 mEq/L
Cl 105 mEq/L	Plt 212 × 10^3^/mm^3^	TSH < 0.1 milliunits/L
CO_2_ 24 mEq/L	WBC 9.5 × 10^3^/mm^3^	FT4 3 ng/dL
BUN 22 mg/dL	Polys 65%	INR 2.7
SCr 1.1 mg/dL	Bands 2%	
Glu 109 mg/dL	Lymphs 30%	

**Table 2:** Learning objectives for ICS.

**Patient Case-1** **Following ICS, the Student Should Be Able to:**
1. Identify, recall, and list the signs and symptoms of CHF and their presentation
2. Identify atrial fibrillation rhythm using telemetry
3. Review home medications and provide initial medication reconciliation
4. Identify the diversity, equity, inclusion (DEI) factors at play, with relevance to the case
5. Use the SBAR communication technique to communicate with the physician and the nurse
6. Display empathy, while providing accurate information
7. Relate the atrial fibrillation presentation to the patient profile, and laboratory values, and identify the underlying pathophysiology (amiodarone-induced T3/T4 change leading to hyperthyroidism)
8. Recall that hyperthyroidism may lead to atrial fibrillation
9. Use their current knowledge of the Vaughan Williams classification to recommend a possible drug for the treatment of the emergent atrial fibrillation
10. Note the effect of “transitions of care” (from ER to admission to discharge) and explain the effect of each change on the Pharmacist Patient Care Process (PPCP). For example, figure out that drug administration, dosage form, or the dose may change.
**Patient Case-2** **Following ICS, in addition to the Learning Objectives described for Patient Case-1, the student should be able to:**
1. Identify rhythms for ventricular tachycardia, Torsades de pointes, and ventricular fibrillation.
2. Explain the underlying pathophysiology and etiology for each of these arrhythmias based on the patient case, the laboratory values, and symptoms. For example, recall the potential of hypokalemia to cause electrical derangement.
3. Explain the role of furosemide as a possible drug-induced this clinical scenario.
4. Recommend a possible therapeutic plan

**Table 3:** Learner performance on ICS pre-and post-tests.

Test Question	Pretest Number of Correct Responses N (%)	Post-test Number of Correct Responses N (%)	Change in Score (%)
Write two symptoms of CHF	34 (50.0)	62 (91.2)	41.2
Which of the following is true regarding atrial fibrillation?	9 (13.2)	8 (11.8)	−1.4
Which of the following may cause atrial fibrillation?	62 (91.2)	60 (88.2)	−3.0
Which of the following arrhythmias is depicted by the image above? (QR widening was shown)	62 (91.2)	66 (97.1)	5.9
For the image in question 4 above, which of the following is true?	68 (100)	68 (100)	0
Which of the following arrhythmias is shown in the image below? (Ventricular tachycardia was shown)	38 (55.9)	63 (92.6)	36.7
Mean percent correct responses	66.9%	80.2%	+13.3%

**Table 4:** Learners’ perception of ICS and its ability to integrate pharmacology and pathophysiology with clinical sciences.

**Area 1: Did ICS Enhance Learner Integration of Pathophysiology and Pharmacology with Clinical Decision Making?**					
Question Statement	SA N (%)	A N (%)	N N (%)	D N (%)	SD
The review lecture prior to the simulation was helpful.	48 (55.8)	34 (39.5)	3 (3.5)	1 (1.2)	0 (0)
The introduction to patient monitor and its components prior to the simulation was helpful.	54 (62.8)	32 (37.2)	0 (0)	0 (0)	0 (0)
The simulation helped me learn the pathophysiology of arrhythmias.	42 (48.8)	31 (36.1)	11 (12.8)	2 (2.3)	0 (0)
The simulation made me interested in clinical application.	58 (67.4)	22 (25.6)	5 (5.8)	1 (1.2)	0 (0)
The simulation made the content seem more relevant to me.	54 (62.8)	24 (27.9)	8 (9.3)	0 (0)	0 (0)
**Area 2: Did ICS Enhance Student’s Experience of Integration–Connecting the Dots between Pharmacology and Pathophysiology and the Clinical Setting?**					
Question statement	SA N (%)	A N (%)	N N (%)	D N (%)	SD
The simulation helped me identify different types of arrhythmias based on the ECG findings.	44 (51.2)	33 (38.4)	4 (4.7)	5 (5.8)	0 (0)
The simulation helped me learn about adverse events associated with antiarrhythmic medications.	32 (37.2)	48 (55.8)	3 (3.5)	2 (2.3)	1 (1.2)
The simulation helped me learn the importance of medication reconciliation and the impact of diversity factors on the same.	42 (48.8)	35 (40.7)	7 (8.1)	2 (2.3)	0 (0)
The simulation enhanced my clinical decision-making process, especially in the context of diversity-related factors.	47 (54.7)	31 (36.1)	6 (7.0)	1 (1.2)	1 (1.2)
The simulation improved my communication skills related to the members of the health care team.	38 (44.2)	35 (40.7)	11 (12.8)	2 (2.3)	0 (0)
The simulation improved communication skills between the team and the patient.	37 (43.0)	27 (31.4)	17 (19.8)	5 (5.8)	0 (0)
The case realistically simulated a situation I may encounter in clinical practice.	49 (57.0)	33 (38.4)	4 (4.7)	0 (0)	0 (0)
**Area 3: Assessing the Impact of ICS**					
Question statement	SA N (%)	A N (%)	N N (%)	D N (%)	SD
The simulated case was challenging enough to intrigue my interests.	47 (54.7)	34 (39.5)	4 (4.7)	0 (0)	1 (1.2)
I have enjoyed the simulation.	47 (54.7)	32 (37.2)	6 (7.0)	1 (1.2)	0 (0)
I would like more simulation activities in other courses.	48 (55.8)	27 (31.4)	9 (10.5)	2 (2.3)	0 (0)

Abbreviations: SA, strongly agree; A, agree; N, neutral; D, disagree; SD, strongly disagree.

## Data Availability

Not applicable.
